# From genes to drugs: targeting Alzheimer’s with circadian insights

**DOI:** 10.3389/fnagi.2025.1527636

**Published:** 2025-03-26

**Authors:** Zekun Li, Xiaohan Li, Lei Su, Zibo Zhang, Hongmin Guo, Yihao Ge, Fang Dong, Feng Zhang

**Affiliations:** ^1^Department of Rehabilitation Medicine, The Third Hospital of Hebei Medical University, Shijiazhuang, China; ^2^Department of Radiotherapy, Affiliated Hospital of Hebei University, Baoding, China; ^3^Metabolic Diseases and Cancer Research Center, Hebei Medical University, Shijiazhuang, China; ^4^Department of Clinical Laboratory Medicine, The Third Hospital of Hebei Medical University, Shijiazhuang, China

**Keywords:** clock genes, Alzheimer’s disease, machine learning, candidate diagnostic biomarkers, immune cell infiltration analysis

## Abstract

**Background:**

Alzheimer’s disease (AD) is a typical neurodegenerative disease that presents challenges due to the lack of biomarkers to identify AD. A growing body of evidence highlights the critical role of circadian rhythms in AD.

**Methods:**

The differentially expressed clock genes (DECGs) were identified between AD and ND groups (non-demented controls). Functional enrichment analysis was executed on the DECGs. Candidate diagnostic biomarkers for AD were screened by machine learning. ROC and nomograms were constructed to evaluate candidate biomarkers. In addition, therapeutics targeting predictive biomarkers were screened through the DGIdb website. Finally, the mRNA–miRNA network was constructed.

**Results:**

Nine genes were identified through the DECG analysis between the AD and ND groups. Enrichment analysis of nine genes indicated that the pathways were enriched in long-term potentiation and circadian entrainment. Four clock genes (GSTM3, ERC2, PRKCG, and HLA-DMA) of AD were screened using Lasso regression, random forest, SVM, and GMM. The diagnostic performance of four genes was evaluated by the ROC curve. Furthermore, the nomogram indicated that ERC2, PRKCG, and HLA-DMA are good biomarkers in diagnosing AD. Single-gene GSEA indicated that the main enrichment pathways were oxidative phosphorylation, pathways of neurodegeneration-multiple diseases, etc. The results of immune cell infiltration analysis indicated that there were significant differences in 15 immune cell subsets between AD and ND groups. Moreover, 23 drugs targeting HLA-DMA and 8 drugs targeting PRKCG were identified through the DGIdb website.

**Conclusion:**

We identified three predictive biomarkers for AD associated with clock genes, thus providing promising therapeutic targets for AD.

## 1 Introduction

Alzheimer’s disease (AD) is a destructive neurodegenerative disorder with a rising global incidence, imposing a significant economic burden on healthcare systems (Scheltens et al., 2021; Zheng and Wang, 2025). As the predominant form of dementia, AD manifests as progressive cognitive decline, memory impairment, and behavioral alterations (Mangal and Ding, 2022; Liu et al., 2024). Mounting evidence suggests that neuropathological changes precede clinical symptoms in AD patients by several decades (Chen and Zhang, 2022; Xu et al., 2024). Therefore, a comprehensive understanding of AD pathogenesis and the identification of novel genes for early diagnosis and treatment are urgent issues that demand attention.

The circadian rhythm serves as an intrinsic biological clock in the human body, forming an approximately 24-h cycle (Xiao et al., 2023). Disruption of the circadian rhythm is a common characteristic observed in neurodegenerative disorders such as AD (Amidfar et al., 2023; Leng et al., 2019; Chen et al., 2024). Impairments in circadian rhythm and sleep patterns among AD patients include fragmented sleep, heightened arousal at night, and reduced daytime activity levels, which can exacerbate AD-related pathologies (Zhang et al., 2025). Studies in mice and humans have shown fluctuations in levels of Aβ and tau proteins in interstitial or cerebrospinal fluid during the sleep/wake cycle, with peaks during the active phase (Kang et al., 2009; Holth et al., 2019; Ma et al., 2016; Roh et al., 2014; Xie et al., 2013; Nguyen Ho et al., 2024). Circadian disruption has also been associated with immune system dysregulation, which can lead to neuronal injury and cognitive decline in AD (He et al., 2023; Weng et al., 2024). Because the circadian rhythm is regulated by a transcription–translation feedback loop involving clock genes (Schrader et al., 2024), identification of clock gene alterations is crucial for further investigations into AD pathogenesis. Therefore, we aim to explore the relationship between clock genes and AD through bioinformatics analysis in the study.

Recently, research on AD has focused on multimodal neuroimaging and deep learning frameworks (Upadhyay et al., 2024b). Upadhyay et al. conducted a comprehensive review of deep learning methods for AD classification, highlighting the potential for integrating structural MRI (sMRI) and functional MRI (fMRI) data (Upadhyay et al., 2024a). Similarly, a study proposed a 3D deep learning model for early AD diagnosis using sMRI and PET imaging, achieving an accuracy of 91.84% (Raza et al., 2024). These new methods can improve the accuracy of AD diagnosis. In contrast to these approaches, our study uniquely combines machine learning with circadian biology to identify biomarkers associated with clock genes. Instead of previous studies that mainly relied on neuroimaging data, we used gene expression profiles to discover new diagnostic markers. This comprehensive approach not only improves diagnostic accuracy but also provides insights into the molecular mechanisms underlying AD progression.

In this study, we utilized a variety of bioinformatics methods and machine learning algorithms to explore the clock genes in AD, with the goal of identifying potential early predictive biomarkers for AD patients and understanding the pathological mechanisms involved. The findings of this study can potentially facilitate the discovery of diagnostic markers for AD. In addition, compared to previous studies (Bacalini et al., 2022; Niu et al., 2022) focusing on single-clock genes (e.g., BMAL1 or PER1), our study integrated multiple machine learning algorithms to prioritize biomarkers (ERC2, PRKCG, and HLA-DMA) with higher diagnostic specificity (AUC > 0.7).

## 2 Materials and methods

### 2.1 Data acquisition

Two Alzheimer’s disease microarray datasets (GSE132903 and GSE122063) were searched on the GEO datasets. The GSE132903 dataset (97 AD patients and 98 non-demented controls) was derived from the GPL10558 platform of the Illumina Human HT-12 v4 expression beadchip. The GSE122063 dataset (12 AD and 11 controls) was derived from the GPL16699 platform of Agilent-039494 SurePrint G3 Human GE v2 8x60K. All samples from the two different databases were obtained from human brain tissue. Details of the datasets are shown in Supplementary Table S1. The clock genes were obtained from the GSEA website.

### 2.2 Recognition of differentially expressed genes (DEGs)

To recognize DEGs between AD and ND groups (non-demented controls), a difference analysis was executed on the GSE132903. The DEGs were selected based on a *p*-value of <0.05 and an absolute log-fold change (FC) > 0.5. The packages “ggplot2” and “pheatmap” were then utilized to draw heatmaps and volcano maps. DEGs and clock genes were intersected to gain differentially expressed clock genes.

### 2.3 Enrichment analysis (EA)

EA of differentially expressed clock genes was carried out utilizing Gene Ontology (GO) and Kyoto Encyclopedia of Genes and Genomes (KEGG) annotations.

### 2.4 Machine learning

Four machine learning algorithms were utilized to screen candidate diagnostic biomarkers for AD: LASSO regression, random forest (RF), support vector machine recursive feature elimination (SVM-RFE), and Gaussian mixture model (GMM). LASSO regression was performed with *λ* selected via minimum mean squared error. Random forest utilized 10-fold cross-validation. SVM-RFE used 5-fold cross-validation. GMM was optimized using the Bayesian Information Criterion. The intersection genes of these four machine learning algorithms had been identified as candidate hub genes for AD diagnosis.

### 2.5 Receiver operating characteristic evaluation (ROC)

The expression of candidate diagnostic biomarkers in the ND group and AD group was compared. In addition, the diagnostic value of each candidate biomarker was evaluated, and ROC was drawn. The area under the ROC curve (AUC) is then calculated with a 95% confidence interval (CI) to estimate the diagnostic value.

### 2.6 Construction of nomogram

A nomogram was built using the rms R package. The diagnosis value of the genes was verified by measuring AUC.

### 2.7 Gene enrichment analysis (GSEA) of hub genes

The GSEA analysis was performed. We calculated the *p*-value for each gene set, and *p-values* < 0.05 were regarded as significant enrichment. Subsequently, the significantly enriched gene sets were visualized.

### 2.8 Analysis of immune cell infiltration

ssGSEA is widely used to evaluate the type of immune cells in the microenvironment. In this study, the ssGSEA algorithm was used to analyze the data of AD patients and quantify the relative proportion of 28 kinds of infiltrating immune cells. The box plot showed a comparison of the differential expression of immune cells between the two groups. The relationship between hub diagnostic biomarkers of AD and the most significantly different and highly expressed immune cells was also investigated.

### 2.9 Drug prediction analysis

Targeted drugs for hub genes were screened through the DGIdb website (https://dgidb.org/).

### 2.10 Construction of mRNA–miRNA networks

We used miRWalk to forecast key miRNAs targeting hub differentially expressed clock genes. The enrichment analysis of CC, MF, BP, and biological pathways of miRNA was performed with FunRich software. In addition, mRNA–miRNA networks with key miRNAs (≥2 differentially expressed clock genes) and hub differentially expressed clock genes were drawn with cytoscape3.10.1.

## 3 Results

### 3.1 Identification of DEGs between AD and ND groups

**Figure 1:**
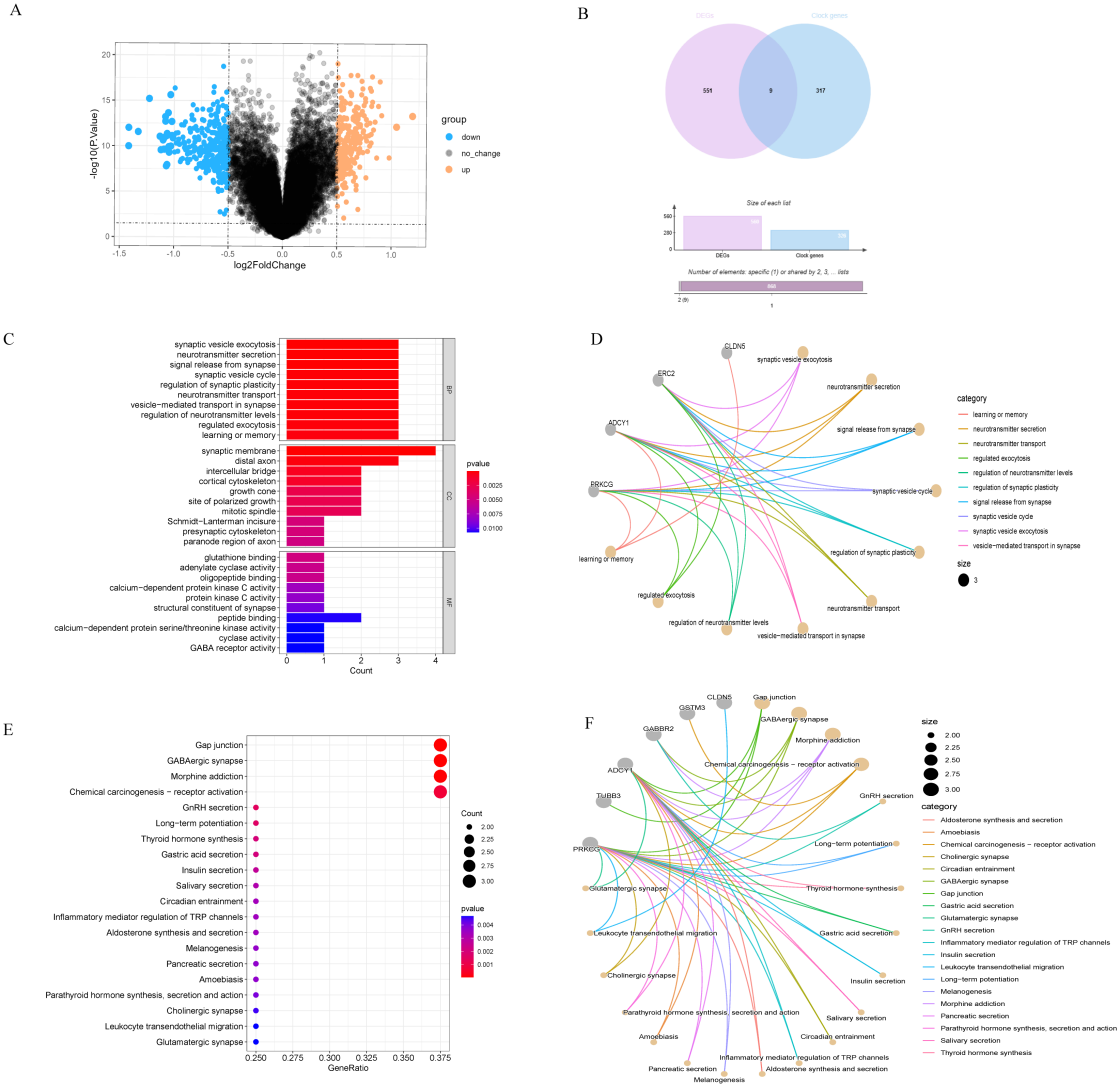
Identification of DEGs in AD patients versus ND and enrichment analysis. **(A)** The volcano map indicated the genes expressed significant differentially between AD and ND groups; orange signified the upregulated genes, and blue signified the downregulated genes. **(B)** Venn diagram of the DEGs and clock genes: purple represented the DEGs, and blue represented the clock genes. **(C,D)** GO EA of intersection nine genes. **(E,F)** KEGG EA of nine genes.

Five hundred and sixty DEGs between the AD and ND groups were identified, of which 323 were upregulated and 237 were downregulated. All DEGs were shown in the volcano map (Figure 1A), with upregulated genes marked orange and downregulated genes marked blue. A total of 326 clock genes were extracted from the GSEA website. By Venn analysis of DEGs and clock genes, nine intersection genes were obtained (Figure 1B). These genes were selected for further analysis due to their potential role in circadian rhythm regulation and AD pathology.

### 3.2 Functional enrichment analysis

In this study, GO and KEGG were used for functional EA of nine intersection genes. The biological process (BP) of GO term analysis indicated that the DECGs in AD were primarily enriched in neurotransmitter secretion, synaptic vesicle exocytosis, etc. In terms of the cellular component (CC) of GO term analysis, the differentially expressed clock genes were mostly located in the synaptic membrane, distal axon, and intercellular bridge. Concerning molecular function (MF) analysis, the results indicated that peptide binding, glutathione binding, and adenylate cyclase activity were the most relevant items of the DECGs (Figures 1C,D). KEGG analysis displayed that the DECGs were primarily enriched in the gap junction, GABAergic synapse, long-term potentiation, circadian entrainment, aldosterone synthesis and secretion, melanogenesis, pancreatic secretion, parathyroid hormone synthesis, secretion and action, cholinergic synapse, and glutamatergic synapse (Figures 1E,F). These pathways were closely associated with AD occurrence and development.

### 3.3 Recognition of hub genes by machine learning

**Figure 2:**
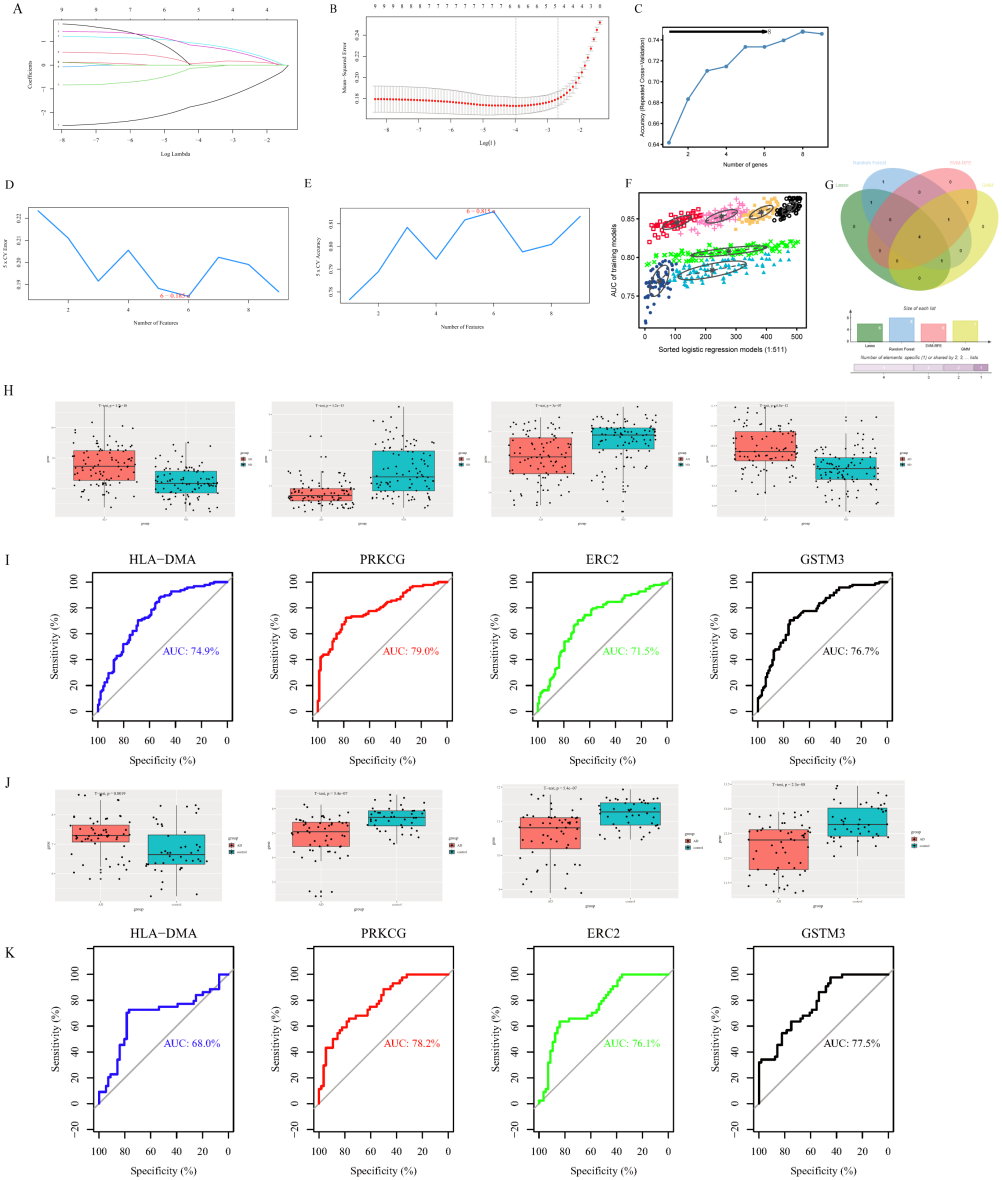
Identification of potential diagnostic biomarkers of clock gene-related AD through machine learning approaches and validation of hub gene diagnostic efficacy. **(A)** Path chart of the LASSO coefficients for intersection genes. Every curve showed the locus of every intersection gene, with the ordinate being the value of the gene, the bottom abscissa being log (*λ*), and the top abscissa being the number of non-zero intersection genes in the model at various time points. **(B)** LASSO regression curve. The diagnostic biomarkers (*n* = 6) were identified by the LASSO. The optimum λ value was determined by 10 times cross-validation. **(C)** The random forest algorithm. **(D,E)** SVM-RFE algorithm screened nine intersection genes to determine the suitable group of feature genes. In the end, eight genes were selected as the optimal feature genes. (F) The GMM classifier determined the average accuracy of a single characteristic gene among the seven combinations. (G) The hub genes were obtained from the LASSO, random forest algorithm, SVM-RFE, and GMM. Validation of hub genes for diagnostic efficacy. **(H)** The levels of HLA-DMA, PRKCG, ERC2, and GSTM3 in the AD group and control group in the GSE132903 dataset. **(I)** The ROC curve displayed the diagnostic performance of the hub gene in the GSE132903 dataset. **(J)** The expression levels of HLA-DMA, PRKCG, ERC2, and GSTM3 in the AD group and control group in the GSE122063 dataset. **(K)** The ROC curve displayed the diagnostic performance of the hub gene in the GSE122063 dataset.

To further simplify the identification of important characteristic variables of the DECGs, Lambda.min and lambda.1se were 0.0187464 and 0.06895639, respectively, and six genes (ERC2, GABBR2, TBL1X, GSTM3, HLA-DMA, and PRKCG) were identified by lambda-based LASSO regression analysis (Figures 2A,B). According to the random forest method, the gene importance score was obtained, and eight candidate genes (PRKCG, GSTM3, HLA.DMA, TBL1X, GABBR2, TUBB3, ERC2, and CLDN5) were identified after the gene was sequenced in order of importance (Figure 2C). The SVM-RFE method found six genes (PRKCG, HLA.DMA, ADCY1, GSTM3, ERC2, and CLDN5) with the minimum error and maximum accuracy following 100 times (Figures 2D,E). As measured by the GMM classifier, the average accuracy of one feature gene in seven combinations was 0.8772354 (Figure 2F). Finally, the common genes (GSTM3, ERC2, PRKCG, and HLA.DMA) were obtained by the four methods (Figure 2G). These genes were selected as hub genes due to their consistent identification across multiple machine learning algorithms.

### 3.4 Property of hub genes to diagnose AD

The level of four hub genes in the AD and ND groups was compared in the GSE132903 training dataset, and the level of four genes displayed statistically remarkable differences between the two groups (Figure 2H). ROC curves were built to evaluate the diagnosis specificity of every gene. Furthermore, we computed the AUC and 95%CI for every project. The results are as follows: HLA-DMA (AUC 0.749, CI 0.681–0.827), PRKCG (AUC 0.79, CI 0.727–0.853), ERC2 (AUC 0.715, CI 0.642–0.788), GSTM3 (AUC 0.767, CI 0.701–0.833) (Figure 2I). In the validation dataset (GSE122063), the expression of four genes displayed statistically remarkable differences between the two groups (Figure 2J). The AUC of four genes was computed in the GSE122063 validation dataset, and the results showed that the AUC of these four genes was slightly higher (Figure 2K). GSTM3 showed inconsistent expression trends between the training dataset (GSE132903) and the validation dataset (GSE122063). This discrepancy might arise from cohort heterogeneity (e.g., differences in disease stages or genetic backgrounds) or technical variability (e.g., batch effects on microarray platforms). In the future, a large sample size and multi-center cohorts are needed to verify the role of GSTM3 in AD.

### 3.5 Establishment and verification for nomogram diagnostic mode

**Figure 3:**
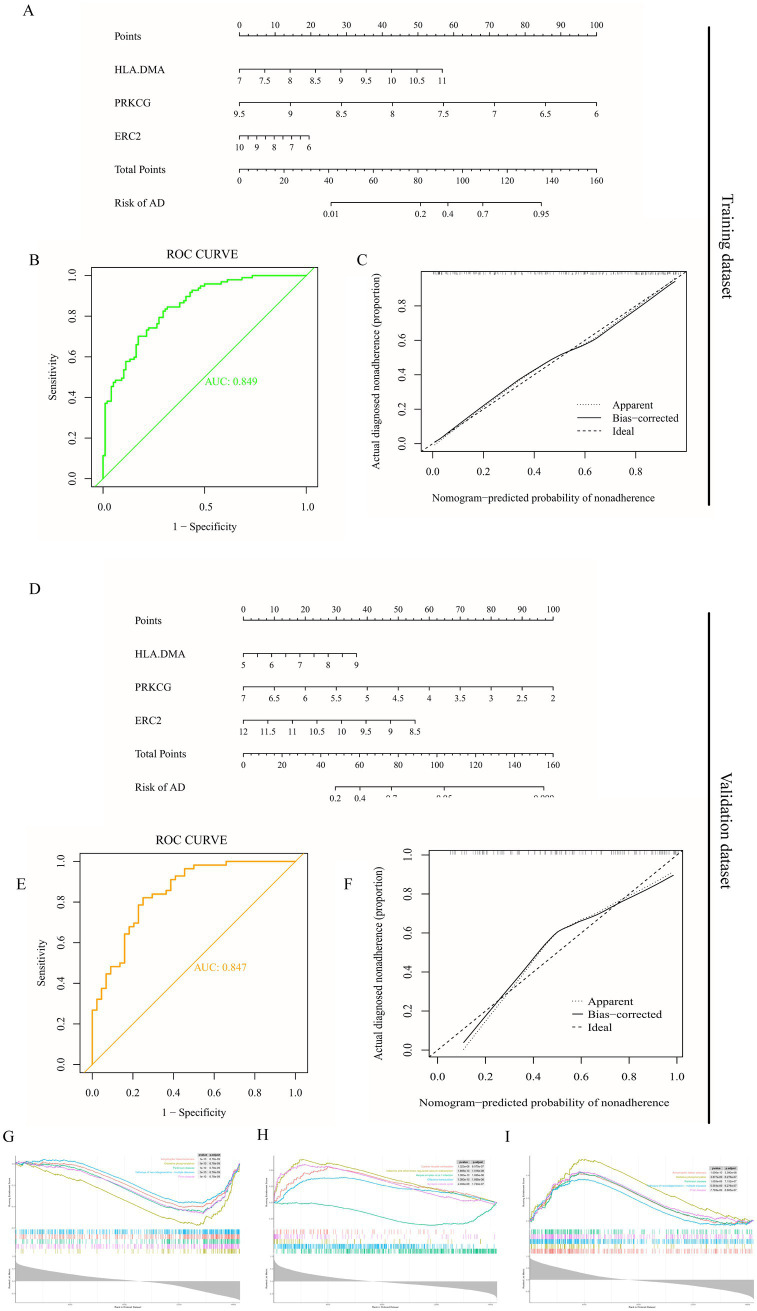
Establishment and verification of nomogram diagnostic model and GSEA enrichment analysis. **(A)** Nomogram model diagram on account of the level of three hub genes in the training dataset (GSE132903). **(B)** ROC curves display the diagnostic property of hub genes in the training dataset. **(C)** Calibration curve displays the predicted performance of the nomogram in the training dataset. **(D)** Nomogram diagram on the account of the level of three hub genes in the validation dataset (GSE122063). **(E)** ROC curves display the diagnostic performance of hub genes in the validation dataset. **(F)** Calibration curve displays the predicted performance of the nomogram in the validation dataset. **(G)** Enrichment results of HLA-DMA. **(H)** Enrichment results of PRKCG. **(I)** Enrichment results of ERC2.

A nomogram model was established for AD diagnosis using three hub genes (HLA-DMA, PRKCG, and ERC2) (Figure 3A). The ROC curve displayed that the combined nomogram model had the highest predictive power compared to other single biomarker models (Figure 3B). Calibration curves were utilized to assess the predictive power of the nomogram model in training datasets. The correction curve showed a small error between the exact and estimated risk for Alzheimer’s disease, showing that the nomogram model was more accurate in forecasting Alzheimer’s disease (Figure 3C). The nomogram was also established in the validation dataset (Figure 3D), and the ROC curve in the validation dataset further verified the effective prediction of the conjoint nomogram model (Figure 3E). The calibration curves for the validation dataset further displayed a precise nomogram model for predicting AD (Figure 3F).

### 3.6 GSEA enrichment analysis of hub genes

We further explored the specific signaling pathways of the three hub genes and investigated the underlying specific mechanisms by which they affect AD progression. GSEA results displayed that the signaling pathways related to the high level of HLA-DMA were oxidative phosphorylation and pathways of neurodegeneration-multiple diseases (Figure 3G). At the same time, the pathways related to the low expression of PRKCG were endocrine and other factor-regulated calcium reabsorption (Figure 3H). The pathways related to the low expression of ERC2 were oxidative phosphorylation and pathways of neurodegeneration-multiple diseases (Figure 3I). These enrichment items were all associated with energy metabolism, suggesting that these three hub genes might be involved in the course of AD by affecting the circadian rhythm and, thus, the body’s metabolism.

### 3.7 Analysis of immune infiltration

**Figure 4:**
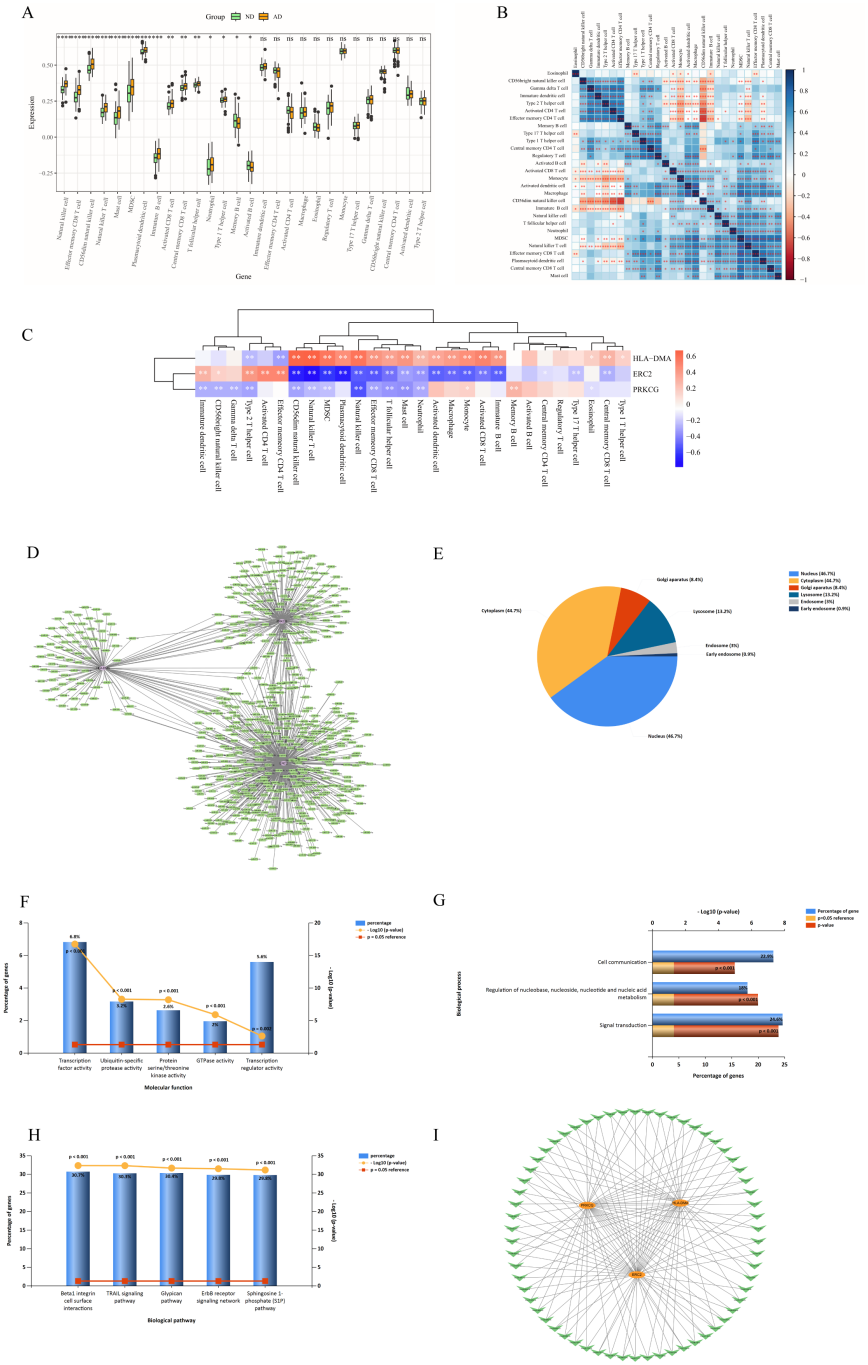
Immune cell infiltration analysis between AD and ND groups. **(A)** Comparison of the percentage of various immune cells between AD and ND groups. **(B)** The correlation of 28 kinds of immune cells in AD was shown by the heatmap. **(C)** Heat map of correlation between three hub genes and immune cells. “*” indicates that the *p*-value is below 0.05, “**” indicates that the *p*-value is below 0.01, and “***” indicates that the *p*-value is below 0.001.

The results of immune cell infiltration analysis indicated that there were significant differences in 15 immune cell subsets between AD and ND groups (Figure 4A). Subsequently, correlations between different immune cells in AD were evaluated (Figure 4B). Moreover, the expression of the three hub genes might influence the level of AD-infiltration immune cell types (Figure 4C). HLA-DMA was positively correlated with immature B cells, activated CD8 T cells, monocytes, macrophages, activated dendritic cells, neutrophils, mast cells, T follicular helper cells, effector memory CD8 T cells, natural killer cells, plasmacytoid dendritic cells, MDSC, natural killer T cells, CD56dim natural killer cells, type 1 T helper cells, central memory CD8 T cells, and eosinophils. ERC2 had a negative relationship with immature B cells, activated CD8 T cells, monocyte, macrophage, activated dendritic cells, neutrophils, mast cells, T follicular helper cells, effector memory CD8 T cells, natural killer cells, plasmacytoid dendritic cells, MDSC, natural killer T cells, CD56dim natural killer cells, central memory CD8 T cells, type 17 T helper cells, and central memory CD4 T cells. There was a negative correlation between PRKCG and neutrophils, mast cells, T follicular helper cells, effector memory CD8 T cells, natural killer cells, MDSC, natural killer T cells, CD56dim natural killer cells, type 2 T helper cells, gamma delta T cells, CD56bright natural killer cells, and immature dendritic cells.

### 3.8 Identification of potential targeted drugs

The DGIdb website was utilized to forecast potential therapeutic drugs for three hub genes. There are 23 potential drugs for HLA-DMA and 8 for PRKCG (additional file: Supplementary Table S2), including vasodilators: fasudil; drugs used to treat AD: BRYOSTATIN, etc. Some of these drugs have been shown to have clinical benefits for AD.

### 3.9 mRNA–miRNA interactive network

The miRNA prediction results of three hub differentially expressed clock genes are shown in Figure 4D. A total of 576 miRNAs were identified, among which 62 miRNAs had a large number of cross-linked genes (differentially expressed clock genes ≥2) (Data Sheet 1). The EA of 576 miRNAs indicated that CC was concentrated in the nucleus (46.7%), cytoplasm (44.7%), Golgi apparatus (8.4%), lysosome (13.2%), endosome (3%), and early endosome (0.9%) (Figure 4E). MF focused on transcription factor activity and so on (Figure 4F). BP is primarily involved in the regulation of nucleobase (Figure 4G). Biological pathways mainly include beta1 integrin cell surface interactions (Figure 4H). Considering the regulatory relationship between miRNA and mRNA, the miRNA–mRNA regulatory network was established utilizing Cytoscape software (version 3.7.2) (Figure 4I).

Figure 4 mRNA–miRNA regulatory network analysis. (A) miRNA prediction results of hub genes. (B) CC EA results of 576 miRNAs via FunRich. (C) MF EA results of 576 miRNAs via FunRich. (D) BP EA results of 576 miRNAs via FunRich. (E) Biological pathways EA results of 576 miRNAs via FunRich. (F) miRNA–mRNA regulatory network, V-type, and circle indicate miRNA and mRNA, respectively.

## 4 Discussion

Alzheimer’s disease is one of the most commonly diagnosed cases of senile dementia in the world (Twarowski and Herbet, 2023). Data from the Alzheimer’s Association Report revealed that approximately 50 million individuals worldwide were afflicted by AD in 2018, with projections estimating a rise to 152 million by 2050 (Zhang et al., 2022). The elevated prevalence of AD imposes a substantial economic and societal burden, presenting a formidable challenge for both individuals and communities (Huang et al., 2024). Current therapeutic approaches to AD encompass cognitive enhancement therapy, management of neuropsychiatric symptoms, and disease-modifying treatments (Zhang et al., 2023). However, several drugs are still under investigation and exhibit limited efficacy (Thakkar et al., 2023). Therefore, exploring new treatment strategies is an urgent task for researchers.

Circadian rhythms, intrinsic 24-h oscillations in charge of regulating daily behaviors and biological processes (Jin et al., 2023), have garnered attention concerning their potential impact on AD. Although the causal relationship between Alzheimer’s disease and circadian rhythm disruption is unclear, there is growing evidence that AD and circadian disruption may interact, with circadian disruption increasing the risk of AD development and AD exacerbating circadian disruption (Rigat et al., 2023; Uddin et al., 2020; McKee et al., 2020). Notably, clock genes have become pivotal roles in regulating circadian rhythms, attracting increased research interest (Zhu et al., 2022). Circadian rhythms are influenced by the dark–light cycle and are chiefly governed via a series of molecular clocks in the suprachiasmatic nucleus (Uddin et al., 2021). Hub clock genes, such as period circadian protein homologs, cryptochromes (CRY1 and CRY2), CLOCK, and BMAL1, coordinate an intricate transcription–translation feedback loop oscillating each 24 h (Takahashi, 2017; Vitaterna et al., 2001). Many studies have highlighted the intimate relation between clock genes and AD pathogenesis (Lananna et al., 2020; Buhl et al., 2019).

In the study, we recognized 560 DEGs between AD and ND groups, with 323 upregulated and 237 downregulated. Through Venn analysis, nine intersection genes (PRKCG, TUBB3, ADCY1, GABBR2, ERC2, GSTM3, TBL1X, HLA-DMA, and CLDN5) were identified from the DEGs and clock genes. These findings hold promise as a foundation for subsequent animal experiments or investigations using human samples, offering valuable insights into the interplay between clock genes and AD progression.

EA of DECGs showed high enrichment levels in BP, including synaptic vesicle, exocytosis neurotransmitter, signal release from synapse, synaptic vesicle cycle, regulation of synaptic plasticity, vesicle-mediated transport in synapse, regulation of neurotransmitter levels, regulated exocytosis, and learning or memory. These processes are closely associated with neurotransmitters, synapses, and learning or memory. The enriched cell components displayed that DECGs were prevalent in the synaptic membrane and distal axon. In terms of molecular function, differentially expressed clock genes are mainly enriched in glutathione binding, structural constituents of synapse, and GABA receptor activity.

KEGG enrichment analysis was mainly enriched in gap junction, GABAergic synapse, long-term potentiation, thyroid hormone synthesis, insulin secretion, melanogenesis, pancreatic secretion, parathyroid hormone synthesis, secretion and action, cholinergic synapse, leukocyte transendothelial migration, glutamatergic synapse, and so on. Notably, in addition to circadian entrainment and synapse-related pathways, other pathways are associated with metabolism and neurotransmission. The high energy requirements of the brain make it very sensitive to modifications in energy metabolism, and metabolic disorders are a hallmark of brain aging, especially in neurodegenerative disorders, for instance, AD. AD patients are often associated with metabolic abnormalities, such as abnormal insulin secretion and parathyroid hormone synthesis (Chen et al., 2023; Patel and Edison, 2024). These metabolic abnormalities further aggravate AD by affecting brain energy metabolism and neuronal health. For example, in AD patients, excessive release of glutamate and overactivation of NMDA receptors lead to increased calcium influx, which in turn causes metabolic disorders (Le Douce et al., 2020). The results of this study emphasize the correlativity between clock genes associated with AD and metabolism and further reveal the correlativity between AD and metabolic disease from the view of clock genes.

Four machine learning methods (LASSO, random forest algorithm, SVM-RFE, and GMM) were utilized to identify differential expression clock genes, and four key genes (GSTM3, ERC2, PRKCG, and HLA.DMA) were obtained. In addition, the expression levels of four genes were evaluated in training and validation datasets, and a clock gene diagnosis model for AD was established and validated. In the construction of the AD model, the three most relevant clock genes were ERC2, PRKCG, and HLA-DMA. Notably, we built a more comprehensive diagnostic nomogram model on account of three hub genes in training and validation datasets, which is more valuable for the diagnosis of AD than independent biomarkers.

We further analyzed the specific signaling pathways of the three hub genes and explored the underlying molecular mechanisms by which they affect AD progression. Single-gene GSEA results show amyotrophic lateral sclerosis, oxidative phosphorylation, Parkinson’s disease, pathways of neurodegeneration-multiple diseases, cardiac muscle contraction, endocrine and other factor-regulated calcium reabsorption, and synaptic vesicle cycle. Previous studies have shown that neurological diseases are closely associated with circadian rhythms. In our study, clock genes associated with AD were concentrated in the pathways related to neurological and metabolic dysregulation, further proving a shared pathway between AD and these systemic disorders from a circadian view.

Previous studies have shown that some clock genes can cause AD through the immune system (Li et al., 2020; Song, 2019). In this study, we found significant differences among activated B cells, activated CD8 T cells, immature B cells, memory B cells, mast cells, MDSC, natural killer cells, natural killer T cells, neutrophils, and other immune cells between AD patients and healthy individuals. HLA-DMA showed a positive correlation with neutrophils, mast cells, T follicular helper cells, effector memory CD8 T cells, natural killer cells, MDSC, natural killer T cells, and CD56dim natural killer cells. However, ERC2 and PRKCG were negatively correlated with neutrophils, mast cells, T follicular helper cells, effector memory CD8 T cells, natural killer cells, MDSC, natural killer T cells, and CD56dim natural killer cells. In summary, these three hub genes exhibited a complex relationship with immune cell infiltration in AD, indicating their potential role in maintaining the balance of the immune response. Therefore, a deeper understanding of the immune mechanisms may enhance the diagnosis of AD and facilitate the development of effective treatments.

This study forecasts potential therapeutic agents for clock genes closely associated with AD patients. These agents include circadian rhythm modulators: bryostatin and bryostatin 1; anti-inflammatory and immunomodulator agents: fasudil, SB220025, UCN-01, PF-562271, tamatinib, and SP-600125; neuroprotective agents: lauroguadine, ingenol mebutate, and quercetin. These potential agents targeting clock genes treat AD through different mechanisms, providing a theoretical basis and feasible direction for subsequent relevant research.

The miRNA plays a key part in the modulation of gene expression and influences the post-transcriptional regulation of genes by binding to mRNA (Fabian et al., 2010). Research has indicated that miRNAs play a crucial part in neurological diseases, including AD (Klyucherev et al., 2022). In our study, we explored the clock genes associated with AD and their corresponding miRNA regulatory networks, revealing the potential mechanisms of these genes in AD pathology. By establishing a miRNA–mRNA network, we obtained miRNAs associated with three key clock genes (HLA-DMA, PRKCG, and ERC2). The interaction of these miRNAs with clock genes may play a crucial part in the occurrence and progress of AD. The construction of miRNA regulatory networks provides a novel perspective for comprehending the intricate gene regulatory mechanism of AD.

EA of 576 miRNAs displayed that they were enriched in β1 integrin cell surface interactions, TRAIL signaling pathway, etc. These pathways play a crucial part in neurodegenerative and metabolic diseases, suggesting that these miRNAs may take part in the pathological course of AD by influencing these key pathways. This study offers important clues for further exploring the specific mechanism of miRNA in AD and is helpful for the future development of miRNA treatment methods for AD. Although this study reveals a potential mechanism of action for miRNAs in AD, further experiments are needed to validate these findings. Future studies can verify the specific regulatory relationship between these miRNAs and clock genes in AD through *in vivo* and *in vitro* experiments. In addition, the in-depth exploration of miRNA expression patterns at different stages of AD and in different patient populations will help to develop personalized treatment regimens.

This study had the following three advantages: (1) This is a novel integration of four machine learning algorithms (LASSO, RF, SVM-RFE, and GMM) to identify AD biomarkers from clock genes, addressing limitations of single-model approaches; (2) It is the first time to combine HLA-DMA, PRKCG, and ERC2 to form a nomogram for AD diagnosis, thus achieving higher diagnostic value; and (3) We systematically explored the role of circadian immune crosstalk in AD. However, this study also had some limitations. First, the dependency on public datasets can induce biases associated with sample collection and management. Second, the variability in gene expression trends across datasets underscores the need for larger, more diverse cohorts to enhance the robustness of our results.

Future studies should emphasize the experimental validation of the identified hub genes and their associated pathways in AD models. Investigating the molecular mechanisms through which these genes influence circadian rhythms and metabolism could provide deeper insights into AD pathology. In addition, exploring the therapeutic potential of the identified drugs in preclinical and clinical studies is crucial. Expanding the scope of immune infiltration analysis to include a broader range of immune cells and their interactions with clock genes may also uncover new therapeutic targets.

## 5 Conclusion

In conclusion, we identified three clock gene-related biomarkers (ERC2, PRKCG, and HLA-DMA) with high diagnostic performance for AD through four machine learning algorithms (LASSO regression, Random Forest, SVM-RFE, and GMM). Functional enrichment analysis revealed that these biomarkers were involved in key pathways such as oxidative phosphorylation and neurodegeneration, highlighting their potential roles in AD pathogenesis. Immune infiltration analysis further uncovered significant differences in 15 immune cell subsets between AD and control groups, suggesting a link between circadian disruption and neuroinflammation. In addition, we identified 23 drugs targeting HLA-DMA and 8 drugs targeting PRKCG, providing a foundation for future therapeutic development. The mRNA–miRNA regulatory network analysis offered novel insights into the post-transcriptional mechanisms underlying AD. These findings not only advance our understanding of the molecular mechanisms linking circadian rhythms to AD but also provide valuable tools for early diagnosis and targeted therapy. Future studies should focus on the experimental validation of these biomarkers and their therapeutic potential in preclinical models.

## Data Availability

The datasets presented in this study can be found in online repositories. The names of the repository/repositories and accession number(s) can be found in the article/Supplementary material.
